# Exploiting Nanotechnologies and TRPV1 Channels to Investigate the Putative Anandamide Membrane Transporter

**DOI:** 10.1371/journal.pone.0010239

**Published:** 2010-04-22

**Authors:** Alessia Ligresti, Luciano De Petrocellis, Dolores Hernán Pérez de la Ossa, Rosario Aberturas, Luigia Cristino, Aniello Schiano Moriello, Andrea Finizio, Mª.Esther Gil, Ana-Isabel Torres, Jesús Molpeceres, Vincenzo Di Marzo

**Affiliations:** 1 Institute of Biomolecular Chemistry, Consiglio Nazionale delle Ricerche (CNR), Pozzuoli, Italy; 2 Endocannabinoid Research Group, Pozzuoli and Naples, Italy; 3 Institute of Cybernetics, Consiglio Nazionale delle Ricerche (CNR), Pozzuoli, Italy; 4 Department of Pharmacy and Pharmaceutical Technology, School of Pharmacy, Complutense University, Madrid, Spain; 5 Department of Pharmacy and Pharmaceutical Technology, School of Pharmacy, Alcalá University, Madrid, Spain; INSERM U862, France

## Abstract

**Background:**

Considerable efforts have been made to characterize the pathways regulating the extracellular levels of the endocannabinoid anandamide. However, none of such pathways has been so argued as the existence of a carrier-mediated transport of anandamide across the membrane. Apart from the lack of molecular evidence for such a carrier, the main reasons of this controversy lie in the methodologies currently used to study anandamide cellular uptake. Furthermore, the main evidence in favor of the existence of an “anandamide transporter” relies on synthetic inhibitors of this process, the selectivity of which has been questioned.

**Methodology/Principal Findings:**

We used the cytosolic binding site for anandamide on TRPV1 channels as a biosensor to detect anandamide entry into cells, and exploited nanotechnologies to study anandamide membrane transport into intact TRPV1-overexpressing HEK-293 cells. Both fluorescence and digital holographic (DH) quantitative phase microscopy were used to study TRPV1 activation. Poly-ε-caprolactone nanoparticles (PCL-NPs) were used to incorporate anandamide, which could thus enter the cell and activate TRPV1 channels bypassing any possible specific protein(s) involved in the uptake process. We reasoned that in the absence of such protein(s), pharmacological tools previously shown to inhibit the “anandamide transporter” would affect in the same way the uptake of anandamide and PCL-NP-anandamide, and hence the activation of TRPV1. However, when masked into PCL-NPs, anandamide cellular uptake became much less sensitive to these agents, although it maintained the same pharmacokinetics and pharmacodynamics as that of “free” anandamide.

**Conclusions:**

We found here that several agents previously reported to inhibit anandamide cellular uptake lose their efficacy when anandamide is prevented from interacting directly with plasma membrane proteins, thus arguing in favor of the specificity of such agents for the putative “anandamide transporter”, and of the existence of such mechanism.

## Introduction

*N*-arachidonoyl-ethanolamine (anandamide, AEA), the first endocannabinoid to be discovered [1] exerts its actions at cannabinoid receptors “on demand” and acts as an autocrine/paracrine mediator. The extent and duration of these actions is determined by AEA extracellular concentration, which, in turn, is controlled by the rate of AEA synthesis and degradation. Since AEA metabolic pathways consist of biosynthetic and hydrolytic enzymes that are intracellularly located, an efficient transport system allowing for the rapid trafficking of AEA through these topographically separated sites was proposed. According to this idea, the uptake of extracellular AEA by the cell might occur as follows: (a) adsorption to the plasma membrane, (b) transmembrane movement, (c) desorption from the plasma membrane, and (d) intracellular trafficking, accumulation, and hydrolysis. However, it is still being argued as to whether the first two steps, i.e. the transport across the membrane, occur via a protein-dependent or independent mechanism. The lack of molecular evidence for the presence of an AEA “membrane transporter” has generated a heated debate on this subject. Although this putative “carrier” has yet to be cloned, indirect biochemical and pharmacological evidence suggests its existence. It has been extensively reported that AEA diffusion through the cell membrane occurs via a saturable, temperature-dependent and selective mechanism [2]–[6]. Furthermore, selective inhibitors of AEA uptake, capable of enhancing AEA effects in vitro and in vivo, and with a pharmacology distinct from that of inhibitors of other AEA inactivation processes, have been developed [4], [7]–[11]. However, the temperature-dependency and saturability typical of AEA uptake could be explained also without the presence of a specific transporter [12],[13]. Moreover, the selectivity of some of the transport inhibitors used so far has recently been questioned ([14], but see also [15]). Furthermore, a contentious has been generated by the different procedures employed for studying the “AEA transporter” [16], [17]. In fact, not only AEA uptake assays might provide different results depending on the experimental conditions [18], but also other factors (i.e. intracellular metabolism, binding to cellular protein or to plastic ware in the absence of albumin) may complicate the measurement of the passage of AEA through the plasma membrane [13].

To help solving this conundrum, here we first decided to study the features of AEA uptake by using an intracellular “biosensor” as a measure of AEA entry into cells, i.e. the activation of the transient receptor potential vanilloid type-1 (TRPV1) channel, which possesses a unique intracellular binding site for AEA in its TM3–TM4 region [19], and responds to the endocannabinoid by gating Ca^2+^ entry into the cell. We performed our experiments in HEK-293 cells overexpressing the recombinant TRPV1 protein (TRPV1-HEK-293 cells), which also possess low levels of expression of the AEA-degrading enzyme, fatty acid amide hydrolase (FAAH) [20], [21]. The activity of TRPV1 was measured both by a conventional fluorescence method to measure intracellular Ca^2+^ and digital holographic (DH) quantitative phase microscopy, which detects cell surface topology perturbations that follow to Ca^2+^ entry. Furthermore, we decided to study the uptake of AEA incorporated into nanoparticles, which are used to facilitate drug delivery to intracellular targets and by-pass any specific and functional interaction with membrane proteins. We reasoned that, if no membrane transporter exists for AEA, one should see no difference in the sensitivity of its uptake to previously reported inhibitors of this process, whether the compound is left free to interact with proteins, or “disguised” into nanoparticles. We expected that, in the absence of a membrane transporter, the pharmacology of AEA uptake by TRPV1-HEK-293 cells would be undistinguishable from that of the uptake of AEA incorporated into poly-ε-caprolactone nanoparticles (PCL-NPs). If this was not the case, this would negate the lack of selectivity of the inhibitors used and hence represent novel evidence for the existence of an AEA transporter.

## Results

### Validation of TRPV1 as a biosensor for the study of AEA cellular uptake

**Figure 1:**
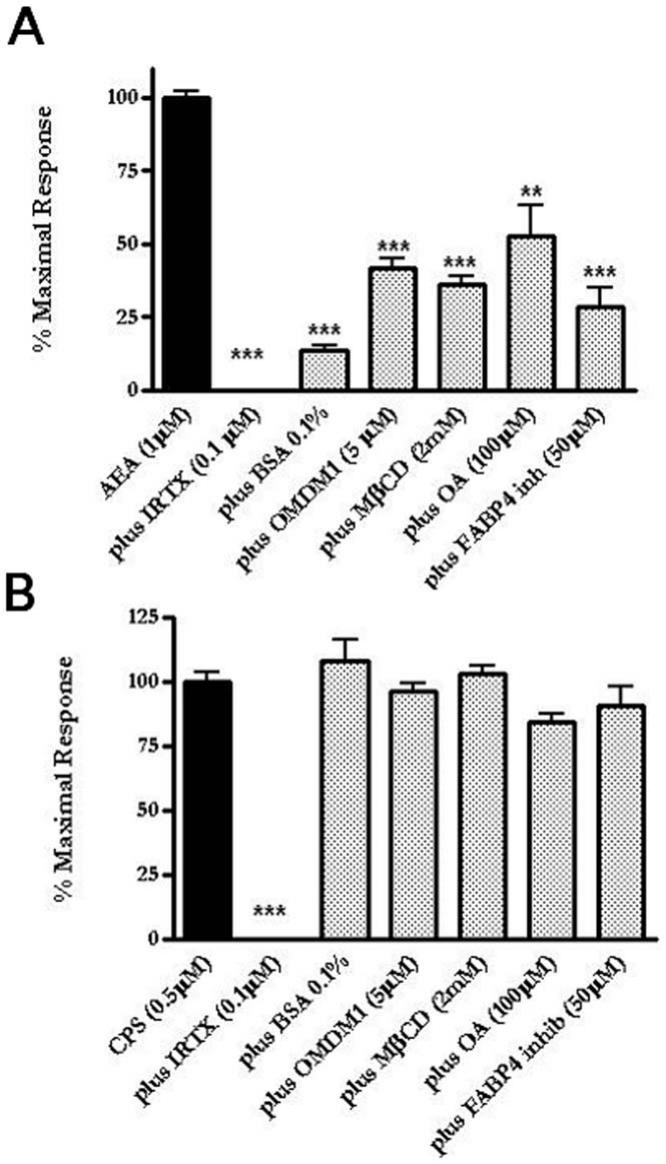
TRPV1 activation as biosensor for the study of anandamide (AEA) cellular uptake. (**A**) AEA-induced elevation of [Ca^2+^]_i_ in intact HEK-293 cells over-expressing the human recombinant TRPV1 receptor was significantly reduced by I-RTX (a selective TRPV1 antagonist) as well by a series of compounds that selectively affect membrane transport processes such as bovine serum albumin) BSA (which sequesters AEA from the extracellular medium); OMDM-1 (a specific inhibitor of AEA cellular uptake with no effect on AEA enzymatic hydrolysis); MβCD (which interferes with AEA cellular uptake by inhibiting the formation of membrane lipid rafts) and oleic acid (OA) and a specific fatty acid binding protein 4-blocker (FABP4 inh) (two inhibitors of the intracellular trafficking of AEA via fatty acid binding proteins). ***p<0.001 compared to AEA+ vehicle (*n* = 3–5). (**B**) Capsaicin (CPS)-induced elevation of [Ca^2+^]_i_ in intact HEK-293 cells over-expressing human TRPV1 receptors was significantly antagonized by I-RTX, but was insensitive to all other compounds that selectively affect membrane transport processes (BSA, OMDM1, MβCD, OA, FABP4 inhib). ***p<0.001 compared to CPS+ vehicle (*n* = 3–5). Data are expressed as percent of the maximal response observed with either AEA (1 µM, 72.0±1% of the effect of ionomycin, 4 µM, see also Fig. 6A) or CPS (76±8% of ionomycin, 4 µM). Each bar indicates means ± sem of at least 3 independent experiments.

In order to validate the use of TRPV1 activation as a biosensor of AEA cellular uptake, irrespective of the mechanism of this latter process, we compared the effect of several pharmacological tools previously shown to interfere with AEA cellular uptake on AEA- and capsaicin-induced elevation of intracellular Ca^2+^ as a measure of TRPV1 activity in TRPV1-HEK-293 cells. As previously shown, AEA induced a dose-dependent increase of intracellular Ca^2+^ ([Ca^2+^]_i_), measured with Fluo-4. The effect of AEA (1 µM), apart from the selective inhibitor of TRPV1 channels, 5′-iodo-resiniferatoxin (I-RTX), was significantly reduced by: 1) bovine serum albumin (BSA), which sequesters AEA from the extracellular medium; 2) OMDM-1 (a specific inhibitor of AEA cellular uptake with no effect on AEA enzymatic hydrolysis [9], [22]; 3) methyl-β-cyclodextrin (MβCD) (which interferes with AEA cellular uptake by inhibiting the formation of membrane lipid rafts [23], [24] and 4) oleic acid (OA) and the specific FABP4 inhibitor [(2′-(5-Ethyl-3,4-diphenyl-1H-pyrazol-1-yl)(1,1′-biphenyl)-3-yl)oxy]-acetic acid, two inhibitors of the intracellular trafficking of AEA via fatty acid binding proteins [25], [26] (Fig. 1A). By contrast, the effect of capsaicin, which acts at the same TRPV1 binding site as AEA, but does not compete with AEA for its uptake, and clearly crosses the plasma membrane through a process different from that involved in AEA transport [7], [20], was antagonized by I-RTX, but was mostly insensitive to other compounds that selectively affect only membrane transport processes (Fig. 1B). These findings indicate that elevation of [Ca^2+^]_i_ in intact TRPV1-HEK-293 cells can be used as a biosensor for AEA cellular uptake in these cells. Importantly, over-expression of human recombinant TRPV1 in HEK-293 cells was not accompanied by any significant change in the expression of cannabinoid CB_1_ or CB_2_ receptors or of FAAH, nor of endogenous AEA levels, as compared to wild-type HEK-293 cells. Furthermore, wild-type HEK-293 cells did not respond to either AEA or capsaicin up to concentrations of 10 µM [20], [21].

**Figure 2:**
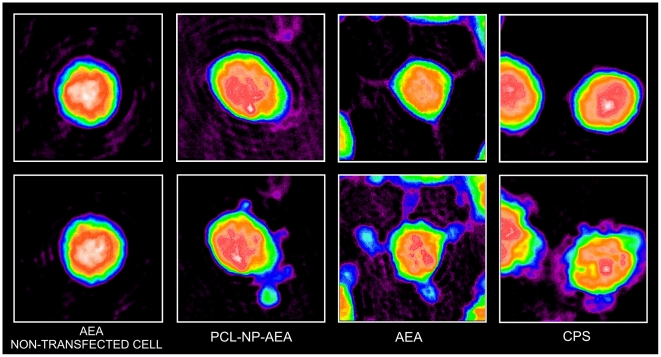
Use of digital holographic (DH) quantitative phase microscopy for the study of AEA uptake. Representative phase images for the effects of anandamide (AEA, 1 µM) in non-transfected HEK-293 cells, and of AEA (1 µM), capsaicin (CPS, 0.5 µM) and PCL-NP-AEA (1 µM) on TRPV1-HEK-293 cell surface topology as observed with digital holographic (DH) quantitative phase microscopy. Apparent membrane extrusions, observed in a TRPV1- and extracellular Ca^2+^-dependent way (see Fig. 3), are shown at ∼200 sec after addition of the drugs (lower panels), whereas the cell surface topology of the same cells is also shown at time 0, prior to addition of the substances (upper panels). See also Videos S1 and S3.

**Figure 3:**
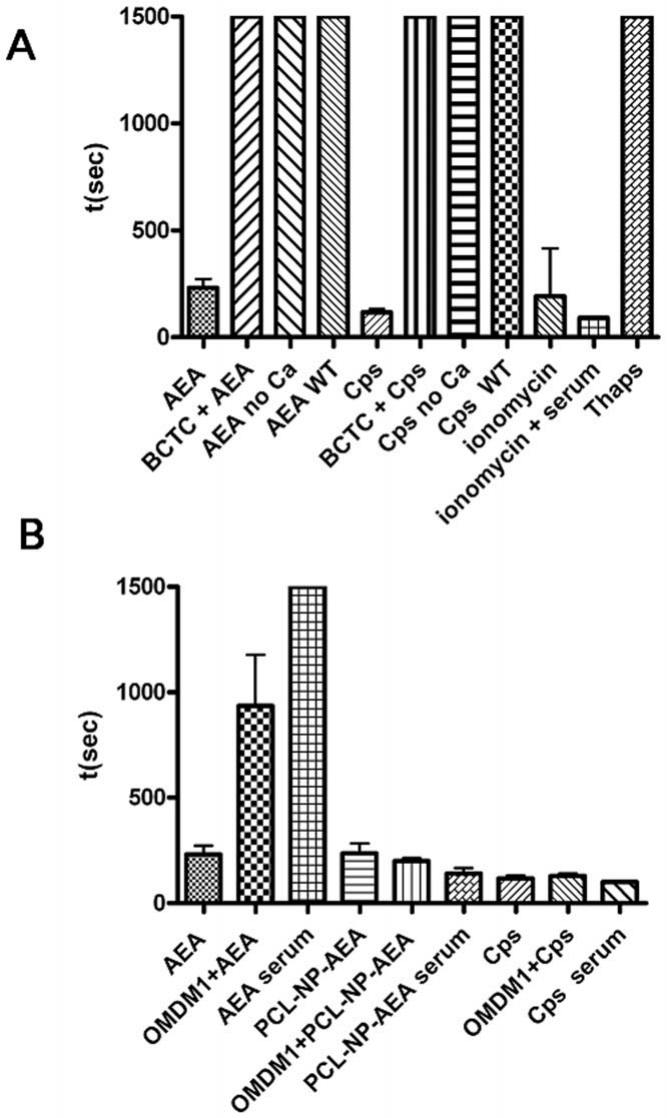
Effects of various drugs or combinations thereof on TRPV1-HEK-293 cell surface topology as observed with digital holographic (DH) quantitative phase microscopy. Bars indicate the mean elapsed time (sec) from the addition of drugs required to observe the first apparent membrane extrusions, with 1500 s as maximum observed elapsed time. (**A**) Effect of anandamide (AEA, 1 µM), *per se* or with the selective TRPV1 antagonist BCTC (0.1 µM), or in the absence of extracellular Ca^2+^ in the medium; of capsaicin (CPS, 0.5 µM) *per se* or with the selective TRPV1 antagonist *N*-(4-Tertiarybutylphenyl)-4-(3-cholorphyridin-2-yl)tetrahydropyrazine -1(2H)-carbox-amide (BCTC, 0.1. µM), or in the absence of extracellular Ca^2+^ in the medium; the calcium ionophore, ionomycin (4 µM), with or without extracellular Ca^2+^ in the medium; and the endoplasmic reticulum Ca^2+^-ATPase inhibitor, thapsigargin (Thaps, 1 µM). The lack of effect up to 1500 s of AEA and CPS in wild-type (WT) HEK-293 cells is also shown. (**B**) Effect of AEA (1 µM), *per se* or in the presence of the AEA uptake inhibitor OMDM1 (5 µM), or of fetal bovine serum (10%) in the medium; of PCL-NP-AEA (1 µM), *per se* or in the presence of the AEA uptake inhibitor OMDM1 (5 µM), or of fetal bovine serum (10%) in the medium; of capsaicin (CPS, 0.5 µM) *per se* or in the presence of the AEA uptake inhibitor OMDM1 (5 µM), or of fetal bovine serum (10%) in the medium. Each bar indicates means ± sem of 3 independent experiments. In (B) the effect of OMDM1 plus AEA on mean elapsed time was significantly different (P<0.01) from that of AEA alone. See also Videos S2 and S4.

We further validated TRPV1 as a biosensor for AEA uptake by using digital holographic (DH) quantitative phase microscopy instead of [Ca^2+^]_i_ measurements. This technique is able to record and then reproduce the light-wavefront scattered from an object, thus allowing the formation of an image of the object itself. This is obtained by recording the “hologram” generated by the optical interference between the light coming from the object and a reference light beam. In particular, DH microscopy was applied to study the effect of AEA on TRPV1-HEK-293 cell surface topology, which consisted in the rapid (200–300 s) induction of membrane extrusions in a TRPV1- and extracellular Ca^2+^-dependent manner (Fig. 2, Video S1, Video S2). This effect of AEA was dramatically delayed by the presence in the culture medium of fetal bovine serum (10%) or by OMDM-1 (Fig. 3A). The Ca^2+^ ionophore ionomycin (induction time 200–400 s), but not the endoplasmic reticulum Ca^2+^-ATPase inhibitor, thapsigargin, which mobilizes intracellular Ca^2+^, also exerted a very similar effect. Capsaicin was capable of inducing a very similar TRPV1- and extracellular Ca^2+^-dependent effect (induction time 100–200 s), although in a way entirely insensitive to BSA and OMDM-1 (Fig. 3B). AEA and capsaicin also caused intracellular light-phase variations, which at present cannot be related to any biochemical effect. A large amount of the collected data will be analyzed in detail in a future paper.

### The encapsulation of AEA into PCL-NPs allows its cellular internalization and cytosolic release

**Figure 4:**
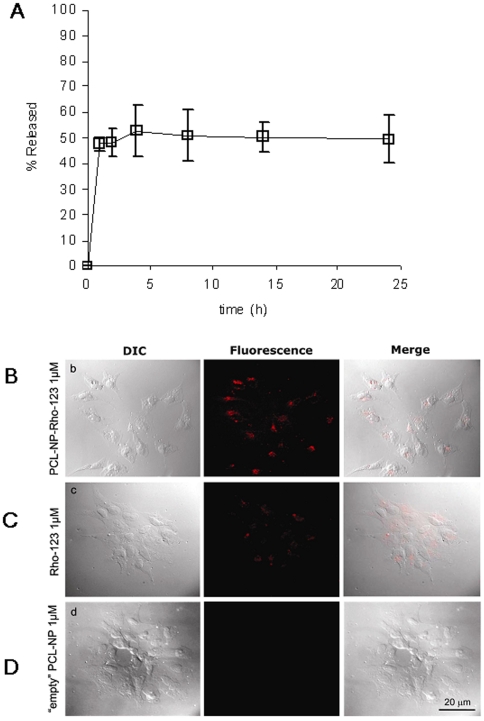
Characterization of anandamide release from PCL-NPs and evidence that PCL-NP rapidly enter HEK-293 cells over-expressing the human recombinant TRPV1 receptor. (**A**) Anandamide release from PCL-NP-AEA at 37°C and at a 99.9% dilution in distilled water (0.5 µl stock suspension in 500 µl water). AEA was measured by HPLC. In a separate experiment, ∼80% of AEA was retained by PCL-NP-AEA incubated for 1 min with the assay buffer used for the experiments of Figs. 1,3. Each bar indicates means ± sem of at least 3 measurements. (**B**, **C**, **D**) Representative images of differential interference contrast (DIC) and fluorescence, and merged images, of cells treated for 1 min at 37°C with Rhodamine-123-loaded nanoparticles (PCL-NP-Rho, 1 µM) (**B**) or “free” Rhodamine-123 (Rho, 1 µM) (**C**). Representative images are also shown for cells treated with “empty” PCL-NP's (**D**).

PCL-NPs were selected for their biocompatibility, lipophilicity, cost-effectiveness and capability to support passive uptake processes, as compared with other polyesters, and were prepared by the nanoprecipitation technique. AEA loading was determined to be 400–500 µg/ml. More than 95% of the drug initially added was entrapped within the polymeric matrix (96.05±1.77%). Particle size was highly reproducible and showed a mean volume (mv) size around 80 nm (83.52±5.3) and 20 nm (21.38±5.7) population width. These parameters were not dependent on the presence of drug (“empty” NP mv was 78.9±3.82 nm and population width 22.95±5.87 nm). Immediate release tests demonstrated that AEA possesses a strong interaction with the polymeric matrix. More than 90% of the drug initially entrapped remains associated to the carrier with a 50% dilution of NP. If diluted to 90% only a small fraction is released (15%). When the dilution is as high as 99%, AEA concentrations in the supernatant (1.83±0.22µg/ml) is comparable to the reported solubility coefficient for THC in aqueous media (2.8 µg/ml) [27] and this is the dilution at which significant drug release takes place, suggesting that solubility in the external medium is a key factor. This also suggests that the interaction between AEA and PCL-NP is not so strong as to prevent AEA release into an aqueous solvent such as the cytosol. However, despite the fact that further dilutions decrease the supernatant concentrations below its solubility coefficient, significant amounts of AEA are still retained by the NP (up to 47%), indicating that a large part of the drug is more stably associated with the NP in the absence of a stronger “binder”. Indeed, the effect of time on drug release at high dilution (99%) showed a two phase profile, with a rapid (but still detectable only after 5 min) release of about 53% of the AEA initially entrapped. Afterwards, the remaining amount of drug remains associated to the polymeric carrier for at least 24 h (Fig. 4A). We also monitored AEA entrapment into PCL-NPs at the final dilution (1µM, 1∶1000) of the stock suspension and with the same buffer used in the uptake/[Ca^2+^]_i_ experiments, by centrifuging the suspension at 15,000×*g* within 2 min from the dilution, and by quantifying AEA release into the supernatant by LC-MS, as previously described [10], [28]. This experiment showed that, under these conditions, most of the AEA was retained into the polymeric matrix, since we could detect in the supernatant only 20.2±2.1% (mean±SD, n = 3) of the total AEA incorporated into the PCL-NPs.

In order to confirm that PCL-NP's rapidly enter TRPV1-HEK-293 cells, we used a microscopy technique, by incubating TRPV1-HEK-293 cells with PCL-NPs encapsulating Rhodamine-123 (PCL-NP-Rho) instead of AEA, and by detecting cytoplasmatic rhodamine under fluorescence microscopy. Rhodamine-123 was efficiently loaded on the PCL-NP approaching 70% of the dye initially added. Particle size was highly reproducible and showed a mean volume (mv) size around 70 nm (76.35±14.35) and 25 nm (26.55±13.78) population width. Immediate release tests evidenced that no rhodamine was released up to 75% NP dilution. PCL-NP-Rho was rapidly and efficiently internalized by cells, as demonstrated by the appearance of cytoplasmic rhodamine within 1 minute (Fig. 4B, C). No effect on cell fluorescence was seen with “empty” PCL-NPs (Fig. 4D).

### The uptake of PCL-NP-AEA has similar pharmacodynamics and pharmacokinetics as “free” AEA uptake but is significantly less sensitive to various inhibitors

**Figure 5:**
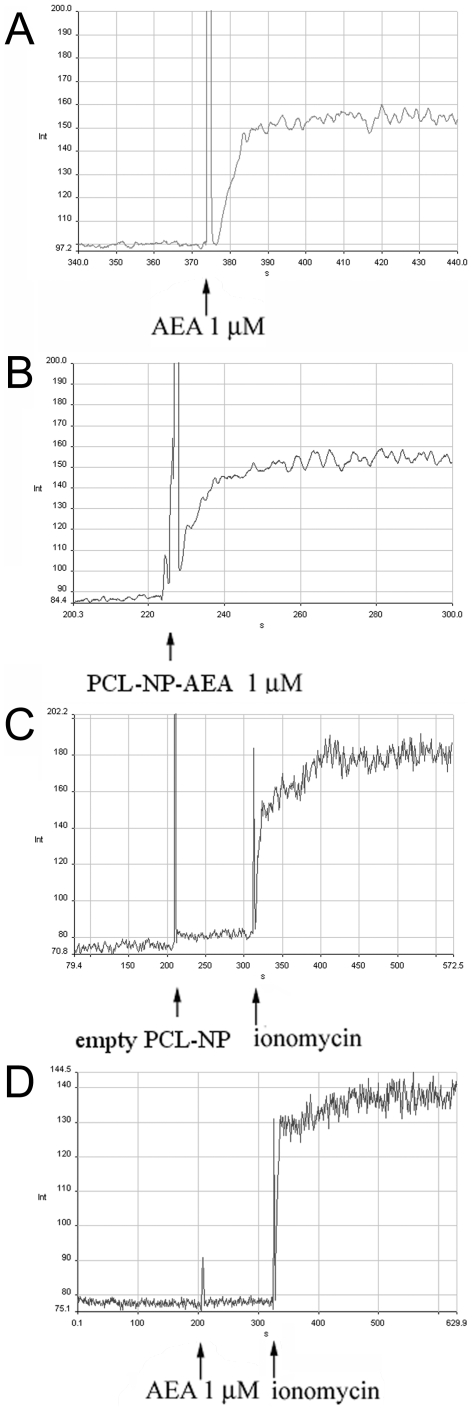
Representative kinetic experiments for the effects on [Ca^2+^]_i_ in intact HEK-293 cells over-expressing the human recombinant TRPV1 receptor. Effect of AEA (**A**) PCL-NP-AEA (**B**) and “empty” PCL-NP's (**C**). The effect of AEA (1 µM) in non-transfected HEK-293 cells is shown in (**D**).

Like “free” AEA, PCL-NP-AEA induced a rapid elevation of [Ca^2+^]_i_ in TRPV1-HEK-293 cells (Fig. 5A,B). Since PCL-NP-AEA needs to be kept at room temperature (25°C) in order to allow stability of nanoparticles, we monitored for 10 days its efficacy as enhancer of [Ca^2+^]_i_ in these cells. The effect of 100 nM PCL-NP-AEA was checked daily and no significant change in efficacy was observed for up to 10 days, unlike what observed with 100 nM free AEA, the effect of which was reduced at day 10 by 50.5±4.3% (mean±SD, n = 3). Importantly, almost no effect on cell fluorescence was seen with “empty” PCL-NPs (Fig. 5C).

**Figure 6:**
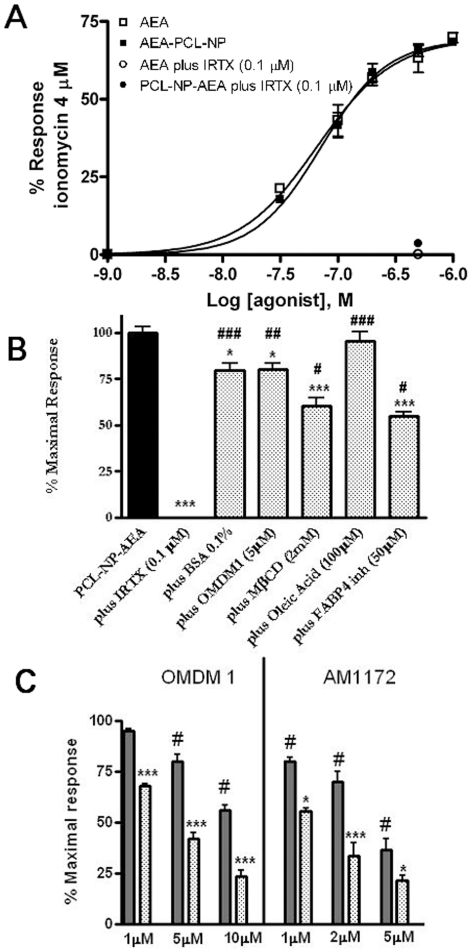
Effect of specific AEA cellular uptake inhibitors on PCL-NP-AEA mediated cellular uptake and [Ca^2+^]_i_ elevation. (**A**) PCL-NP-AEA (▪) exhibited similar potency and efficacy as “free” AEA (□) on [Ca^2+^]_i_ in intact HEK-293 cells over-expressing the human recombinant TRPV1 receptor. The effect is expressed as percent of the effect on Ca^2+^ by ionomycin, 4 µM, and, in both cases, was dose-dependent and TRPV1-mediated because it was abolished by I-RTX (• and ○). (**B**) PCL-NP-AEA-induced elevation of [Ca^2+^]_i_ in intact TRPV1-HEK-293 cells over-expressing the human TRPV1 receptor was significantly less sensitive to pharmacological tools previously used to specifically interfere with AEA cellular uptake or intracellular trafficking (BSA, OMDM1, MβCD, OA, FABP4 inhib). *p<0.05; ***p<0.001 compared to AEA-PCL-NP + vehicle (*n* = 3–5); #, ##, ### p<0.05, p<0.01 and p<0.005 compared with AEA+corresponding inhibitor (see Fig. 1A). (**C**) PCL-NP-AEA effect (full bars) on TRPV1-mediated [Ca^2+^]_i_ was significantly less sensitive to concentrations of the selective AEA uptake inhibitors, OMDM-1 and AM1172, which instead strongly affected the effect of “free” AEA (dotted bars). *p<0.05; ***p<0.001 compared to the corresponding effect of the inhibitor observed using PCL-NP-AEA; # p<0.05 compared with PCL-NP-AEA + vehicle. In (**B**) data are expressed as percent of the maximal response observed with PCL-NP-AEA (1 µM) + vehicle (see panel (**A**)). In (**C**) data are expressed as percent of the maximal response observed with either PCL-NP-AEA (1 µM) + vehicle or AEA (1 µM) + vehicle (see panel (**A**)). Each bar indicates means ± sem of at least 3 independent experiments.

**Figure 7:**
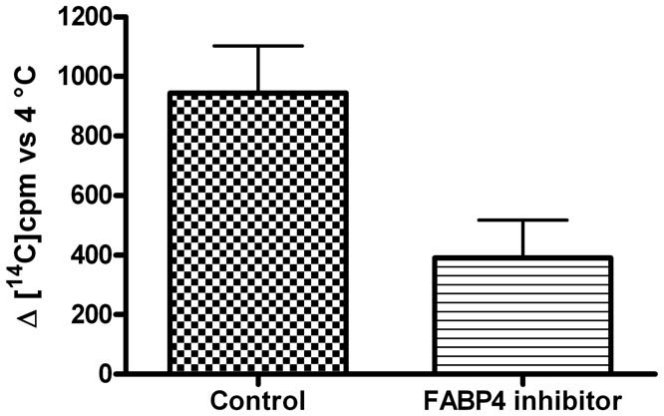
Effect of the fatty acid binding protein-4 (FABP4) inhibitor [(2′-(5-Ethyl-3,4-diphenyl-1H-pyrazol-1-yl)(1,1′-biphenyl)-3-yl)oxy]-acetic acid, 50 µM) on the cellular uptake of [^14^C]-anandamide in a “conventional “uptake assay carried out in TRPV1- HEK-293 cells. The amount (cpm) of [^14^C]-AEA taken up by cells at 37°C subtracted of that taken up at 4° after a 90 sec incubation with the compound is shown on the *y* axis. Each bar indicates means ± sem of 3 independent experiments. The effect of the FABP4 inhibitor was significantly different (P<0.01) from that of vehicle (0.1% methanol).

The effect of PCL-NP-AEA on [Ca^2+^]_i_ in TRPV1-HEK-293 cells was dose–dependent and TRPV1-mediated because it was abolished by I-RTX (Fig. 6A). It exhibited nearly identical kinetics (a Ca^2+^ peak was observed after 10 seconds of exposure, Fig. 5B), potency and efficacy as that of “free” AEA (Fig. 6A). Importantly, however, the TRPV1-mediated, and, hence, AEA uptake-dependent, effect of PCL-NP-AEA on [Ca^2+^]_i_ was significantly less sensitive to all the tools used so far to interfere with AEA cellular uptake or intracellular trafficking. First, it was much less significantly inhibited by BSA (0.1%) (Fig. 6B), an avid binder of “free” extracellular AEA that strongly inhibits “free” AEA effects (Fig. 1A) [21]. This finding represents also further evidence that in PCL-NP-AEA suspension most of AEA is tightly encapsulated into nanoparticles and not free. Next, we found that the effect of PCL-NP-AEA was less sensitive to plasma membrane cholesterol depletion and subsequent lipid raft destabilization by MβCD (Fig. 6B) than “free” AEA (Fig. 1A), although still significantly inhibited. Indeed, also the NP-endocytotic mechanism was shown to rely in part on the formation of lipid rafts [29]–[31], although perhaps not as strongly as AEA cellular uptake [32]. The effect of PCL-NP-AEA was also dramatically less sensitive to oleic acid or the FABP4 inhibitor [(2′-(5-Ethyl-3,4-diphenyl-1H-pyrazol-1-yl)(1,1′-biphenyl)-3-yl)oxy]-acetic acid) (Fig. 6B) than that of “free” AEA (Fig. 1A), thus possibly indicating that NP-mediated delivery and intracellular release renders AEA less available for the binding to FABPs. Since this particular inhibitor had never been tested on AEA uptake [25], we validated its use by showing that it does inhibit the cellular uptake of [^14^C]-AEA in a “conventional” uptake assay carried out in TRPV1- HEK-293 cells (Fig. 7). Finally, and most importantly, the effect of PCL-NP-AEA on TRPV1-mediated [Ca^2+^]_i_ was much less sensitive to concentrations of the selective AEA uptake inhibitors, OMDM-1 [9] and AM1172 [22], which instead potently inhibited the effect of “free” AEA (Fig. 6C). Similar results were obtained when the uptake of PCL-NP-AEA as compared to “free” AEA was studied by using DH microscopy (Fig. 3B). The TRPV1 and, hence, cellular uptake dependent effect of PCL-NP-AEA on plasma membrane topology (induction time 200–300 s), which was still dependent on extracellular Ca^2+^, was not delayed by either BSA or OMDM-1, unlike what observed with “free” AEA (Fig. 3B, Video S3, Video S4).

## Discussion

The lack, to the present date, of any molecular evidence for an AEA membrane transporter has generated a heated debate on its existence, particularly in view of the fact that the lipophilicity of AEA would allow it to cross the plasma membrane simply by passive diffusion. Despite indirect data indicating the presence of a specific and protein-mediated mechanism facilitating AEA transport across the plasma membrane, in a direction according to the gradient of its concentration, and hence possibly mediating also AEA release after its *de novo* biosynthesis, some authors suggested that such process was uniquely facilitated by the intracellular enzymatic hydrolysis of this compound by FAAH [15], [16]. Later, other groups showed that AEA cellular uptake can occur rapidly also in the absence of FAAH [6], [17], [22], although the actual mechanism through which this phenomenon occurs, and whether it is facilitated by membrane or, instead, cytosolic proteins, as suggested by very recent data [25], [26], is yet to be solved. Recent experiments have also indicated that the temperature-requirement of AEA cellular uptake is not sufficient evidence for such process to occur via a “carrier”-mediated mechanism, and that temperature-dependent AEA uptake can occur also in cell “ghosts” or artificial membranes devoid of membrane proteins [34], [35]. Yet, artificial membranes are not like real cells, and the fact that AEA rapid diffusion through membranes can occur in the absence of proteins does not exclude the existence of specific proteins that regulate this process in real life. To date, the strongest evidence in favor of the existence of these proteins comes from experiments using synthetic compounds that are able to interfere with AEA cellular uptake or release without acting on AEA catabolism or biosynthesis. Some of these compounds exhibit a pharmacology clearly different from that of other tools used to inhibit AEA metabolism, such as the FAAH inhibitors, also in vivo [11]. However, any inhibitor is selective until proven otherwise, and the fact that these compounds inhibit AEA cellular uptake does not provide conclusive evidence for the existence of a “transporter”.

**Figure 8:**
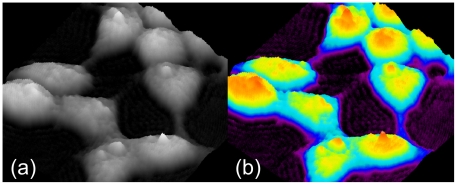
Examples of living cell images, obtained by a digital holographic microscopy (pseudo 3D rendering). (**A**) In the image, each pixel grey-value is a measure of the light phase amount in the corresponding point of the sample; (**B**) codified-colours rendering allows for improved evaluation of phase values.

Since all indirect evidence in favor of the presence of an AEA transporter is debatable, we decided to use, instead, a strategy aimed at gaining, or failing to gain, direct evidence for its absence. We studied, in intact HEK-293 cells, which were previously suggested to possess a putative AEA transporter, but little, if any, FAAH [20], the pharmacology of the cellular uptake of AEA as such or prepared in a way to prevent it from interacting with any plasma membrane protein. The only way to achieve this task was by incorporating AEA into nanoparticles, a strategy widely used to deliver drugs directly to intra-cellular targets bypassing their direct and specific interactions with membrane and intracellular carriers. We reasoned that if, under these conditions, the uptake of AEA was still equally sensitive to more or less selective tools previously shown to inhibit the uptake of “free” AEA, including inhibitors of the putative AEA transporter, on which the suggestion of the existence of such mechanism is mostly based, this would represent compelling evidence against this suggestion. Since different (and, in some cases, even similar) experimental conditions for the study of the uptake of radiolabeled AEA by intact cells, including varying length of incubation of AEA with cells, different types of plastic ware, etc., were shown to lead to different results, we decided here to monitor AEA uptake “while it occurs” by using a biosensor for the entry of AEA into intact cells. This approach allowed us to avoid all the lengthy processing of, and measurements in, fixated cells, which can be done only much after that the uptake has occurred. We exploited the property of TRPV1 channels to be activated by AEA exclusively at an intracellular domain [19] and, subsequently, to cause elevation of [Ca^2+^]_i_, measured by a simple fluorimetric assay, or membrane topology perturbation, measured by DH microscopy (Fig. 8).

We previously used the observation that AEA stimulation of TRPV1-mediated Ca^2+^ elevation in HEK-293 cells is sensitive to some inhibitors of anandamide uptake to suggest that AEA acts on TRPV1 at an intracellular domain [20], a suggestion that was later conclusively demonstrated by using TRPV1 mutants lacking the intracellular domain between the TM3-TM4 α-helices [19]. However, no previous study has ever validated the measure of TRPV1-mediated rapid elevation of [Ca^2+^]_i_, or of plasma membrane perturbation by DH microscopy, as a biosensor for AEA entry into the cell. Therefore, we first proceeded here to fill this gap and, indeed, showed that both measurements are capable to clearly discriminate between the cellular uptake of a fatty acid amide, such as AEA, for which evidence for interaction with a putative transporter exists, and another fatty acid amide, capsaicin, which penetrates the plasma membrane via a mechanism clearly distinct from that of AEA. We next demonstrated that the PCL-NPs efficiently and stably incorporate AEA as well as a fluorescent dye, which they deliver across the plasma membrane to release it directly into the cytosol. Finally, we could carry out two major observations: 1) the kinetics, efficacy and potency of PCL-NP-AEA at activating TRPV1, and hence at entering HEK-293 cells, are undistinguishable from those of “free” AEA – this allowed a direct comparison between the effects of inhibitors of AEA uptake on the two types of preparation; 2) the sensitivity of AEA activation of TRPV1, and hence of AEA uptake, to previously reported inhibitors of this latter process, and, in particular, to two selective inhibitors of AEA uptake, was, instead, significantly different, and dramatically lower when the endocannabinoid was prevented from interacting with plasma membrane and intracellular proteins by its incorporation into PCL-NPs. Instead, the sensitivity of the effects of the two different AEA formulations to TRPV1 antagonists were identical. These data fail to demonstrate the hypothesis of the lack of specificity of the pharmacological tools used here, and in particular of the inhibitors of anandamide cellular uptake, hypothesis that instead would have been supported by the observation of identical sensitivity of PCL-NP-AEA and “free” AEA uptake to these compounds. Therefore, these data represent strong evidence in favor of the opposite thesis, i.e. that these inhibitors do indeed act by preventing the interaction of AEA (but not of PCL-NP-AEA) with a putative membrane carrier. This approach exemplifies the widely accepted Euclidean *reductio ad absurdum*, for which a proposition that cannot be demonstrated as true proves that its contrary is instead true, since a third possibility does not exist (*tertium non datur*). It is worth mentioning that some selective inhibitors of AEA uptake, although not those used in the present study, were previously found to inhibit also AEA interaction with, and release from, plastic ware [36]. However, this phenomenon is unlikely to have biased at least our experiments with fluorimetric detection of intracellular Ca^2+^, which were carried out in a quartz cuvette.

Based on the high loading stability and efficiency of PCL-NP-AEA and on previous reports about the factors affecting NP cell uptake [31], it is reasonable to assume that the PCL-NP-AEA coated by Pluronic F-68 are rapidly internalized by hTRPV1-HEK-293 cells. Hence, the fact that PCL-NP-AEA effects were only weakly modified by inhibitors of intracellular AEA trafficking, which instead efficiently reduced the effects of “free” AEA, is likely the result of endosomal NP escape and AEA release in close proximity to its binding site on TRPV1. Therefore, these results support the innovative idea to carry out the present study with a validated nanoparticle-based approach. However, there was an apparent discrepancy in the AEA retention ability of PCL-NPs upon dilution depending on the method and buffer used to assess this parameter. The original data of release studies at 99.9% dilution (0.5 µl stock suspension in 500 µl water) at room temperature show that ∼47% of the AEA initially added remains associated to NP, whereas the amount was ∼80% when a similar dilution was carried out using the incubation buffer for TRPV1 activity assays. This difference is likely the result of a salting out effect already reported for THC (solubility in water 2.8 mg/l and 0.77 mg/l in 0.15 M NaCl at 23°C) (it should be noted that THC solubility in 0.15 M NaCl is close to the reported value for AEA in 0.16 M PBS 7.4) [27]. Thus, the salting out effect might favor the retention of lipophilic drugs such as AEA by PCL-NPs, and it is very likely that the release of AEA from PCL-NPs was much slower following their dilution in the buffers used to carry out fluorescence and DH microscopy studies than in distilled water. On the other hand, the fact that ∼20% of AEA was potentially released from PCL-NPs prior to the entry of the latter into cells probably explains the residual inhibitory effect of BSA and other pharmacological tools used here on the uptake of PCL-NP-AEA. In other words, the fact that the uptake inhibitors OMDM1 and AM1172, or the FABP4 inhibitor, still slightly reduced the TRPV1-mediated effect of PCL-NP-AEA on Ca^2+^, at concentrations which instead inhibited the effect of “free” AEA almost maximally (note that the maximal inhibition of AEA effect observed here is about 80%), might be due to the release from nanoparticles of some AEA *prior* to the cellular uptake of PCL-NP-AEA, and to the counteraction by these inhibitors of the uptake of this small amount of “free” AEA rather than of PCL-NP-AEA.

In conclusion, we have presented here for the first time data showing that AEA cellular uptake can be studied in living cells using the TRPV1 channel as a biosensor, DH microscopy as a means to investigate TRPV1 activity in alternative to fluorescence, and incorporation of AEA into nanoparticles. Using these novel technologies we reported that negating the direct interaction of AEA with putative plasma membrane proteins, by disguising it into nanoparticles, renders its uptake significantly less sensitive to most known types of modulators of this process. In our opinion, these data strongly suggest that AEA uptake is a process that can be counteracted by selective inhibitors and hence facilitated by specific plasma membrane protein(s), possibly in concert with intracellular trafficking proteins. This work should encourage specific studies aimed at finally identifying and cloning these proteins. However, our data will have to be challenged in the future by continuing to study the effect on nanoparticle-incorporated AEA of yet to be developed tools that specifically interfere with the cellular uptake of AEA.

## Materials and Methods

### PCL-NP synthesis and characterization

Poly-ε-caprolactone (PCL) was obtained from Sigma (Spain) and Pluronic F-68 was from Fluka (Germany). All organic HPLC solvents were from Scharlab (Spain) and water was MilliQ grade (Millipore, USA). Sigmacote from Sigma was used for glassware treatment to reduce drug adsorption. Nanoparticles (NP) were obtained by the nanoprecipitation method from 150 mg polycaprolactone and 5 mg anandamide (AEA) (5 mg/ml). Drug loading was determined by HPLC as previously described [37] from the amount of drug in the supernatants and the total drug amount in the sample. Mean particle size and size distribution of NP was established by laser light diffraction (Microtrac, Leeds and Northrup, St. Petersburg, USA). Immediate drug release studies assessed the retention ability of the polymeric carrier upon variable dilution (from 99.9% to 50%). Drug release behaviour with time was also studied after incubation of 99% diluted NP samples at 37°C for 1, 4, 8, 14 and 24 h.

### Release of AEA from PCL-NP-AEA into the assay medium by LC-MS

PCL-NP-AEA (1 µl) from the stock solution (1 mM) was diluted with 1 ml of Tyrode's solution (145 mM NaCl, 2.5 mM KCl, 1.5 mM CaCl_2_, 1.2 mM MgCl_2_, 10 mM D-glucose, and 10 mM HEPES, pH 7.4), mixed and left at 37°C for 2 min prior to centrifugation at 15,000×*g*. After the centrifugation processes, 100 µl of the supernatant were mixed with 10 pmol of d8-AEA (as an internal standard) and directly analyzed by liquid chromatography–atmospheric pressure chemical ionisation-mass spectrometry (LC–APCI-MS) [10], [28] using a Shimadzu HPLC apparatus (LC-10ADVP) coupled to a Shimadzu (LCMS- 2010) quadrupole MS via a Shimadzu APCI interface. The temperature of the APCI source was 400°C; HPLC column was a Phenomenex (5µm, 150mm×4.6mm) reverse phase column, eluted whit a mixture of Methanol/H_2_O/CH_3_COOH 85∶15∶0.1% (1 ml/min). MS analyses were carried out in the selected ion monitoring in a positive-ion mode. The selected ions correspond to a mass/charge ratio (m/z) of 348.0 and 356.0 (respectively [M-H]^+^ for deuterated and non-deuterated AEA). Quantitative measurements of AEA levels were obtained by analysis of the area ratio between non-deuterated and deuterated AEA.

### Rhodamine-loaded PCL-NP synthesis, characterization and entry into TRPV1-HEK-293 cells

Rhodamine-loaded NP were also prepared by the nanoprecipitation method described above. Rhodamine-123 (Sigma) was added to the polymer acetone solution before addition of this mixture to the water-pluronic mixture. The concentration of the fluorophore was set at 1.0% (w/w) of the polymer as previously reported [38]. Dye loading (%) was determined by UV-Vis spectrometry at 505 nm as described for drug loading determination. Mean particle size and size distribution of NP was established by laser light diffraction (Microtrac, Leeds and Northrup, St. Petersburg, USA). Immediate release studies assessed the retention of the dye by NP upon sequential dilution in PBS (from 50% to 99.9%) in PBS pH 7.4. Rhodamine release with time at 75% dilution in PBS pH 7.4 was determined at 2, 4, 8 and 24 h.

TRPV1-HEK-293 cells were plated overnight on poly-D-lysine coated cover slips and, then, incubated for 1 minute at 37°C in a cell medium containing 1µM of PCL-NP-Rho-123 or “free” Rhodamine-123 or “empty” PCL-NP's. After incubation, cells were washed at room temperature in PBS (Phosphate saline solution, pH = 7.4), fixed with paraformaldehyde 4% for 10 minutes, washed in PBS and mounted using Aquatex Antifade. The cells were studied through Leica DMI6000B inverted digital microscopy. The same cells were observed in bright field by differential interference contrast (DIC), and in fluorescence field under appropriate filter for rhodamine-123 excitation. The images were acquired using digital camera Leica DFC 340FX connected to the microscope and analyzed by LAS AF 2.2.0^©^ software.

### Intracellular calcium assays

This assay was performed as previously described [20], [21]. HEK-293 (human embryonic kidney) cells stably transfected with the human recombinant TRPV1 were obtained as described previously [20], [21] and grown in minimum essential medium (EMEM) supplemented with nonessential amino acids, 10% fetal calf serum, and 2 mM glutamine, maintained under 5% CO_2_ at 37°C plated on 100-mm diameter Petri dishes. The effect of “free” AEA or PCL-NP-AEA on [Ca^2+^]i was determined by using Fluo-4, a selective intracellular fluorescent probes for Ca^2+^. At this aim, on the day of the experiment, cells were loaded for 1 h at room temperature with the methyl ester Fluo4-AM 4 µM; containing 0.04% Pluoronic F-127 (Invitrogen) in minimum essential medium without foetal bovine serum. After loading, cells were washed with Tyrode's solution (145 mM NaCl, 2.5 mM KCl, 1.5 mM CaCl_2_, 1.2 mM MgCl_2_, 10 mM D-glucose, and 10 mM HEPES, pH 7.4) and transferred to the quartz cuvette of the spectrofluorimeter (Perkin-Elmer LS50B; PerkinElmer Life and Analytical Sciences, Waltham, MA) under continuous stirring. Experiments were carried out by measuring cell fluorescence at 25°C (λ_Ex_ = 488nm, λ_Em_ = 516nm) before and after the addition of compounds. Curve fitting (sigmoidal dose-response variable slope) and parameter estimation were performed with GraphPad Prism® (GraphPad Software Inc., San Diego, CA). The efficacy of the agonists was first determined by normalizing their effect to the maximum Ca^2+^ influx effect on [Ca^2+^]_i_ observed with application of 4 µM ionomycin (Sigma). Potency was expressed as the concentration of test substances exerting a half-maximal agonist effect (i.e. half-maximal increases in [Ca^2+^]_i_) (EC_50_), calculated by using GraphPad®.

### Digital Holographic (DH) quantitative phase microscopy

Digital holography is an optical imaging and measurement method, utilizing the features of modern digital devices. Holography, in general, is able to record and then to reproduce the light-wavefront scattered from an object, allowing for the formation of an image of the object itself (“hologram”). The hologram is generated by the optical interference between the light coming from the object and a reference light beam. To record a hologram, a special optical arrangement is required, using a laser as light source. In traditional holography, a special photographic plate is used as recording apparatus. After chemical processing, the resulting hologram, illuminated with a light beam, act as a diffraction grating and provides a replication of the original object wavefront. In DH, the hologram is recorded by a high resolution digital camera and stored as data-file; the wavefront reconstrution is carried out by applying numerical methods to the stored data set. The final output are both an amplitude image and a phase image of the object. Moreover, a number of suitable algorithms allow for numerical post-processing (i.e. focus adjustments, aberrations compensation and more), obtaining enhanced quality intensity images and very accurate quantitative phase-maps of the samples. The phase-map is a prominent feature of DH as it represents, pixel by pixel, the path-lenght measure of the light trasmitted (or reflected) by the object. DH methods are widely used for high accuracy measurements and diagnostic applications in technological and material-science fields, as a non-invasive, advanced metrological tool, and there is growing interest towards its biological applications. Fig. 8a shows a tipical, grey-scale, DH microscope phase-image of living-cells in a Petri dish without any fixation process. A suitable codified-colours rendering can then greatly improve the image evaluation (Fig. 8b).

In conventional optical microscopy, well known contrast-enhancing methods are adopted to overcome the poor visibility of cells observed by bright-field illumination. Such methods, by using special optical configurations, are able to convert light-phase changes into the corrisponding intensity changes, thus achieving a significant enhancement of sample visibility. However, these methods but are not useful for quantitative phase measurements and suffer of some lateral-resolution reduction. In this context, DH has been demonstrated to be instead a very effective, label-free, method for obtaining quantitative phase-images: on living cell samples it allows an impressive imaging enhancement and intracellular refractive index and thickness measurements. Lateral resolution is preserved, while an axial resolution up to 25 nm in thickness can be obtained. Fast events can be analyzed by acquiring a sequence of digital holograms, this being limited only by the frame-rate capability of the used camera. Presently a few commercial DH microscopes are available from specialised providers. As references, a few publications have been selected to take a quick “bird's eye” look at the state of the art on biological applications of DH, and as a useful starting point for further searching. In [39], [40], [41], [42] an introduction to some implementations of DH microscopy, can be found: explanations on basic optical configurations and on numerical data processing methods are provided. The remarkable (quantitative) imaging capability of DH is demonstrated, both by single images, of fixed or living cells, and by movies (available on line). Examples of DH microscopy applications are reported in the following references: 1) in [43] the DH method was investigated for detection of lipid-particle dissemination in living cell cultures, as an alternative procedure to the previous, time-consuming, methods which need fixing and dying the cells; 2) in [44] an improved implementation of DH demonstrated its ability in revealing and mapping subcellular motion inside a tumor spheroid, responding to anticancer drugs; 3) in [45] a DH method enabled accurate measurements of red blood cells membrane fluctuations, adopting a procedure to separately measure the cell refractive index and its thickness. This procedure is an useful advance towards the solution of an important task of DH, since it is worthwhile mentioning that the phase measurement achievable by this technique actually represents the path-lenght variations of the light interacting with the sample. In a transparent object, such variations are modulated by both refraction-index and thickness variations of the object itself. In unpredictable cases, they can be concurrent and, consequently, added in the final measured phase value. In such a case, it might become mandatory to decouple their contributes. Studies are ongoing in defining valid paradigms for specific imaging and/or quantitative applications of the DH methods in biology: this requires efforts in comparative studies using a strict interdisciplinar cooperation between experts in life-science and optics.

In the present work, we used an experimental version of a DH microscope, in transmission configuration, for time-lapse investigations on morphological changes of living-cells, before and after the addition of compounds. The light source was a 532 nm (green) laser. Attention was paid to reduce the light intensity impinging on the cells to minimize thermal or photochemical effects. However all pilot tests carried out on each measured sample did not show any evidence of such effects. Cells were placed in small plastic Petri dishes in EMEM with or without 10% FBS (2 ml) and directly analysed with the DH microscope, before and after addition of drugs in DMSO (max volume 2 µl), at room temperature (24°C±2). A large number of measurements were made, each consisting of two holograms series acquisitions: first, as a control, a 10 minutes sequence of the untreated cells was acquired; then a sequence was acquired during the addition of compounds. The frame rate was of 10 frames per second. After the computational process, the phase-maps collections were used for movies assembling, in order to analyse the cell topology variations, and the related timings.

### Cellular uptake of radiolabeled AEA by TRPV1-HEK-293 cells

The effect of FABP4 inhibitor [(2′-(5-Ethyl-3,4-diphenyl-1H-pyrazol-1-yl)(1,1′-biphenyl)-3-yl)oxy]-acetic acid) on the uptake of [^14^C]-AEA in human embryonic kidney cells over-expressing the human recombinant TRPV1 protein (TRPV1-HEK-293) was studied as previously described [6], by using 2 µM (10,000 cpm) [^14^C]-AEA. Cells were plated to 6-wells culture plates at a density of 1–2×10^6^ cells/well, preincubated for 30 minutes with 50 µM of the FABP4 inhibitor in EMEM at 37°C and subsequently incubated with [^14^C] anandamide for 90 sec. After incubation, residual [^14^C]-AEA in the incubation medium after extraction with CHCl_3_/CH_3_OH 2/1 (by volume), was determined by scintillation counting of the lyophilized organic phase and used as a measure of the amount of anandamide taken up by cells. Specific AEA uptake was determined by subtracting uptake at 4°C from uptake at 37°C. Data are expressed as cpm of [^14^C]-AEA taken up by cells at 37°C subtracted of that taken up at 4°.

### Statistical analyses

All determinations were performed at least in triplicate. Statistical analysis of the data was performed by analysis of variance at each point using ANOVA followed by the Bonferroni's test.

## Supporting Information

Video S1 Effects of anandamide (AEA, 1 microM) on TRPV1-HEK-293 cell surface topology as observed with digital holographic (DH) quantitative phase microscopy.
(1.41 MB MOV)

Video S2 Effects of anandamide (AEA, 1 microM) plus OMDM-1 (5 microM) on TRPV1-HEK-293 cell surface topology as observed with digital holographic (DH) quantitative phase microscopy.
(1.64 MB MOV)

Video S3 Effects of PCL-NP-AEA (1 microM) on TRPV1-HEK-293 cell surface topology as observed with digital holographic (DH) quantitative phase microscopy.
(2.33 MB MOV)

Video S4 Effects of PCL-NP-AEA (1 microM) plus OMDM-1 (5 microM) on TRPV1-HEK-293 cell surface topology as observed with digital holographic (DH) quantitative phase microscopy.
(1.21 MB MOV)
